# Salty Taste Acuity Is Affected by the Joint Action of αENaC A663T Gene Polymorphism and Available Zinc Intake in Young Women

**DOI:** 10.3390/nu5124950

**Published:** 2013-12-05

**Authors:** Hwayoung Noh, Hee-Young Paik, Jihye Kim, Jayong Chung

**Affiliations:** 1 Department of Food and Nutrition, College of Human Ecology, Seoul National University, Seoul 151-742, Korea; E-Mails: hyoung1012@gmail.com (H.N.); hypaik@snu.ac.kr (H.-Y.P.); 2 Research Institute of Human Ecology, College of Human Ecology, Seoul National University, Seoul 151-742, Korea; 3 Department of Medical Nutrition, Graduate School of East-West Medical Science, Kyung Hee University, Yongin 446-701, Korea; E-Mail: kjhye@khu.ac.kr; 4 Department of Food and Nutrition, College of Human Ecology, Kyung Hee University, Seoul 130-701, Korea

**Keywords:** salty taste acuity, αENaC A663T gene polymorphisms, zinc intake

## Abstract

Salty taste perception affects salt intake, of which excess amounts is a major public health concern. Gene polymorphisms in salty taste receptors, zinc status and their interaction may affect salty taste perception. In this study, we examined the relationships among the α-epithelial sodium channel (αENaC) A663T genotype, zinc intake, and salty taste perception including salty taste acuity and preference in healthy young adults. The αENaC A663T genotype was determined by the PCR-restriction fragment length polymorphism in 207 adults. Zinc intake was examined by one 24-h recall and a two-day dietary record. Salty taste acuity and preference were determined by measuring the salty taste recognition threshold and the preferred salinity of beansprout soup, respectively. Men had significantly higher thresholds and preferences for salty taste than women did (*p* < 0.05). In women, the salty taste threshold was significantly lower in the highest tertile of available zinc intake than in the lowest tertile (12.2 mM and 17.6 mM, respectively, *p* = 0.02). Interestingly, a significant inverse association between available zinc intake and salty taste threshold was found only in women with αENaC AA homozygotes (β = −0.833, *p* = 0.02), and no such association was found in T663 allele carriers. The salty taste preference was not associated with the αENaC A663T genotype or available zinc intake in either sex. In conclusion, our data suggest that gene-nutrient interactions between the αENaC A663T genotype and available zinc intake play a role in determining the salty taste acuity in young women.

## 1. Introduction

Excessive table salt intake is a well-known risk factor for the development of hypertension, cardiovascular disease and renal disease [1,2]. Recent evidence predicted that even a modest reduction (3 g/day) of salt intake could substantially reduce the incidence of cardiovascular disease and related medical costs [3]. However, despite continued efforts to reduce salt intake, excessive salt consumption remains a major public health concern in many populations. In Korea, the average daily sodium intake is more than three times the WHO recommendation (<2000 mg/day) [4,5]. Salty taste perception such as salty taste acuity and salty taste preference are considered important determinants of salt intake [6]. For example, Kim *et al*. (2009) [7] reported associations between elevated salty taste thresholds, elevated preference for salt in broth, and elevated consumptions of salty foods among adolescents. Similarly, the taste threshold for salt in tomato soup was higher for the high salt intake group compared to the low salt intake group [8]. Therefore, studies to identify factors that contribute to salty taste perception are needed to establish effective strategies for the reduction of salt intake.

Genetic polymorphisms in salty taste receptors may contribute to the variability of salty taste perception among individuals. The epithelial sodium channel (ENaC), which consists of at least three subunits (α-, β- and γ-), is a major sodium-specific taste receptor in mammals [9]. Amiloride, an ENaC blocker, alters sodium currents in taste cells and inhibits taste response to sodium chloride [10,11,12]. Further, the lack of αENaC in taste cells led to a complete loss of salt attraction and sodium taste responses in mice [13]. Several different polymorphisms in the ENaC gene have been identified in humans [14]. In particular, a single A to G nucleotide substitution in the αENaC gene that produces an amino acid change of alanine to threonine at residue 663 (αENaC A663T) has been found to be common in diverse ethnic groups [15]. A recent study has found that a genetic polymorphism in αENaC is associated with differences in amiloride-sensitive taste responses to sodium chloride in mice [16]. In addition, the αENaC A663T gene polymorphism was found to affect the risk for developing hypertension, suggesting functional changes in the activity of the ENaC ion channel due to the αENaC A663T polymorphism [14,15]. The relationship between αENaC A663T polymorphism and salt taste perception, however, has not been studied previously.

Zinc deficiency has been associated with the impairment of taste perception [17]. Zinc is required for the enzymatic function of gustin (carbonic anhydrase VI), which is present in parotid saliva. The decreased secretion of salivary gustin is related to reduced or distorted taste and smell function [18]. Previous studies have reported that experimental zinc depletion significantly decreased salty taste perception [19], and zinc supplementation enhanced taste acuity in elderly subjects [20] and in patients with taste disorders [21,22]. Furthermore, studies have shown that zinc enhances the sodium-transfer activity of ENaC [23], suggesting a potential interaction of zinc status and ENaC genotypes on salt taste perception.

Previous studies have suggested that genetic polymorphisms in taste receptors influence the perception of basic tastes such as bitter and sweet taste [24,25,26]. Little is known, however, as to the association between the genetic polymorphisms in salty taste receptors and salty taste perception. As mentioned earlier, the αENaC A663T gene polymorphism is one of main polymorphisms found in the αENaC gene and has been linked to changes in the ENaC activity. Therefore, we hypothesized that the αENaC A663T gene polymorphism, zinc status and their interaction may affect salty taste perception. To test this hypothesis, in the present study, we investigated the relationships among αENaC A663T gene polymorphism, zinc status and salty taste perception including salty taste acuity and preference in young adults.

## 2. Subjects and Methods

### 2.1. Study Design and Subjects

We recruited 207 healthy young adults (104 men and 103 women), aged 20–29 years, who did not smoke or take any medications or dietary supplements, from a university campus. Participants visited our laboratory twice. At the first visit, salty taste acuity and preference were measured by sensory evaluation, and dietary intake data were collected using a 24-h recall method. At the second visit, fasting blood samples and anthropometric data were obtained. Additionally, subjects submitted two-day dietary intake records at the second visit. All procedures were approved by the Institutional Review Board of Seoul National University (IRB NO.0812/001~001). Written informed consent was obtained from all participants.

### 2.2. Genotyping

The αENaC A663T genotype of each subject was determined using the PCR-restriction fragment length polymorphism method, as described by Sugiyama *et al*. [15]. Total genomic DNA was extracted from 200 µL whole blood using QIAamp DNA blood mini kits (Qiagen, New York, NY, USA). A 50 ng sample of genomic DNA was used as the template in a PCR reaction using an MJ mini gradient thermal cycler (Bio-Rad, Hercules, CA, USA). PCR primer sequences were: 5′-TCCCTCTCCAGCCTTGACAGC-3′ (sense) and 5′-TTGCTTCCCCTCCACACATCA-3′ (antisense) [15]. PCR amplification was carried out with an initial denaturation at 95 °C for 5 min, followed by 40 cycles of 94 °C for 30 s, 58 °C for 30 s, 72 °C for 45 s and a final extension at 72 °C for 5 min. PCR products (251 bp) were verified by electrophoresis on a 2% agarose gel with ethidium bromide. PCR products were digested with 10 units AciI (New England Biolabs, Ipswich, MA, USA) at 37 °C for 16 h, separated on an 8% acrylamide gel and stained with ethidium bromide. Bands were visualized using the Gel Doc XR system (Bio-Rad, Hercules, CA, USA).

### 2.3. Sensory Evaluation

Taste acuity refers to the range of taste stimulus that a person is able to recognize and is usually assumed from taste threshold assessment [27]. In this study, salty taste acuity was determined by measuring the salty taste recognition threshold (herein after referred as salty taste threshold), which was the level at which a salty stimulus can be identified using a modified “up-down” procedure [28]. Fifteen sodium chloride test solutions from 0.85 mM to 68.38 mM were prepared. Samples were offered to subjects in order of salt concentration, from lowest to the highest. If the subject detected a salty taste in two consecutive samples, the lower concentration of the two samples was recorded as the salty taste threshold 1. In the second trial, samples were offered to subjects in the reverse order, from the highest to the lowest concentration. If the subject reported no detection of a salty taste for two consecutive samples, the concentration of the immediately preceding solution (with a concentration higher than that of the two samples) was recorded as salty taste threshold 2. The average of the two salty taste thresholds was used for the subsequent analyses.

Salty taste preference refers to the attitude or response of a person toward salty taste [29]. We determined the salty taste preference by measuring the preferred concentration of sodium chloride in a clear soup made with soybean sprouts, a common Korean dish. The soup sample was made with 1 L filtered water and 400 g bean sprouts that were boiled for 20 min and cooled to a temperature of 50–60 °C. The sodium concentration of soups without added salt was 6.84 mM (0.04%). We asked subjects to season the soup samples with salt to suit their taste. We then collected these samples and measured the sodium concentration twice with a salt-meter (TDS, Daeyoon Scale Industrial Co., Seoul, Korea). All subjects were asked to abstain from drinking alcoholic beverages for 12 h and to avoid intake of any other food or brushing their teeth at least for two hours before the sensory evaluation.

### 2.4. Dietary Assessment

Dietary intake data were collected in one 24-h recall and a two-day dietary record. At the first visit, all subjects were asked about what they ate on the previous day using the 24-h recall method, and they were shown in a face-to-face interview how to record their dietary intakes for the two-day record. The subjects were instructed to record the amounts of all of the food and beverages they consumed in two inconsecutive days, including one weekday and one weekend day. In total, three-day intake data for two weekdays and one weekend day were collected. To help improve the accuracy of quantifying the amount of food consumed, 100% scaled pictures of rice and soup bowls, side dishes and a cup were provided. The dietary records were checked by trained staff for completeness of information, and daily energy and nutrient intake were calculated using the Korean Nutrition Society Database by DS24 program [30]. For energy intake adjustment, daily zinc intake was expressed as mg zinc intake per 1000 kcal energy intakes. Because dietary phytate significantly affects the bioavailability of dietary zinc [31], available zinc intake was estimated based on the phytate-to-zinc molar ratio using a standard algorithm [32,33,34]; the zinc availability factors were set at 0.5, 0.3 and 0.15 for phytate-to-zinc molar ratios of <5, 5–15 and >15, respectively.

### 2.5. Anthropometric and Biochemical Assessment

Anthropometric characteristics such as height, weight and body mass index (BMI) were measured by trained staff using a BMI measuring device (BSM 330, Biospace Co. Ltd., Seoul, Korea). BMI was calculated as weight (kg) divided by height squared (m^2^). Serum zinc levels were analyzed by atomic absorption spectrometry (AAS 600, Perkin Elmer, Waltham, MA, USA), and blood hemoglobin, and serum albumin levels were measured using an automatic analyzer (ADVIA 2400, Bayer Diagnostics, Tokyo, Japan). Biochemical assessment was conducted by a clinical laboratory (Eone Reference, Laboratory, Incheon, Korea).

### 2.6. Statistical Analysis

All data analyses were conducted using SAS 9.3 (SAS Institute Inc., Cary, NC, USA). All data were presented as the mean and standard error of the mean (SEM). Non-normally distributed variables were natural logarithmically transformed. Differences in general characteristics were examined using Student’s *t*-test. The chi-square test (χ^2^-test) was used to examine the difference in the distribution of zinc status and αENaC A663T genotypes between men and women. The distributions of salty taste threshold and preference between men and women were examined by the Cochran-Manetel-Haenszel test. Trends of change in salty taste threshold and preference among αENaC A663T genotypes and across tertiles of zinc intake were examined by the generalized linear model (GLM). Multiple regression analyses were conducted to identify the effect of zinc intake on salty taste threshold by the αENaC A663T genotype. After subjects were stratified into two groups according to available zinc intake, then the differences of salty taste threshold between subjects with each zinc level in αENaC AA homozygotes and subjects with αENaC T663 allele carriers were examined by the GLM. In addition, the interaction of available zinc intake and the αENaC A663T genotype on salty taste threshold in women was also examined by the GLM. All GLM and regression models were adjusted for BMI as a potential confounder.

## 3. Results

The mean age of the study subjects was 23.6 years and the mean BMI, serum albumin and hemoglobin were all within the normal range in both men and women (Table 1). Sodium intake was significantly higher in men (4114.2 mg) than in women (3071.5 mg). Total and available zinc intakes were not significantly different between men and women. The percentage of subjects whose zinc intake was below the estimated average requirements (EAR) was 56.3% in women and 38.5% in men (*p* < 0.01). Serum zinc concentration was significantly lower in women (13.6 µM) than in men (14.4 µM) (*p* = 0.04), but the percentages of subjects with serum zinc deficiency were not significantly different between men (12.5%) and women (10.7%).

The salty taste threshold was significantly lower in women (15.8 ± 0.9 mM) than in men (19.3 ± 1.2 mM) (*p* = 0.02), and the salty taste preference was also significantly lower in women (75.4 ± 3.0 mM) than in men (86.0 ± 4.0 mM) (*p* = 0.03) (Table 1). There was a significantly positive correlation between salty taste threshold and preference (*r* = 0.26, *p* < 0.001). The salty taste threshold was positively correlated with the sodium intake (*r* = 0.18, *p* < 0.01), but no correlation was found between the salty taste preference and the sodium intake (*r* = 0.07, *p* = 0.34) (data not shown).

**Table 1:** General characteristics of study subjects ^a^.

	Men	Women	*P* ^e^
	(*n* = 104)	(*n* = 103)	
Age (years)	23.6 ± 2.2	23.6 ± 2.3	0.87
BMI (kg/m^2^)	23.0 ± 2.1	20.9 ± 1.8	<0.001
Albumin (g/dL)	4.9 ± 0.3	4.8 ± 0.2	0.02
Hemoglobin (g/dL)	15.8 ± 1.0	13.4 ± 0.9	<0.001
Energy intake (kcal/day)	2116.2 ± 449.3	1630.0 ± 355.8	<0.001
Sodium intake (mg)	4114.2 ± 113.8	3071.5 ± 78.8	<0.001
Sodium intake (mg/1000 kcal)	1960.6 ± 45.2	1907.7 ± 41.8	0.39
Zinc intake (mg/1000 kcal)			
Total zinc	4.2 ± 0.9	4.4 ± 1.1	0.22
Available zinc ^b^	1.8 ± 0.5	1.9 ± 0.6	0.19
<EAR ^c^	40 (38.5)	58 (56.3)	0.01
Serum zinc (μM)	14.4 ± 2.8	13.6 ± 2.5	0.04
Serum deficiency ^d^	13 (12.5)	11 (10.7)	0.68
Salty taste threshold (mM)	19.3 ± 1.2	15.8 ± 0.9	0.02
Salty taste preference (mM)	86.0 ± 4.0	75.4 ± 3.0	0.03

^a^ Data are expressed as means ± SEM or *n* (%); ^b^ Available zinc intake was estimated based on the phytate-to-zinc molar ratio using a standard algorithm [32,33]; ^c^ EAR (Estimated average requirements) for zinc: Men—8.1 mg/day, Women—7 mg/day; ^d^ Men—<11.3 μM, Women—<10.7 μM [35]; ^e^ Differences between men and women were examined by Student’s *t*-test or χ^2^-test.

**Figure 1:**
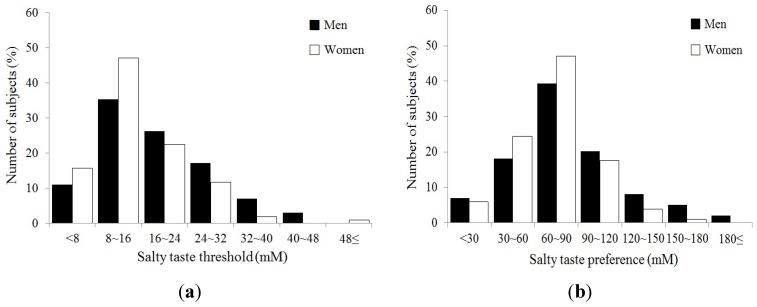
Distribution of salty taste threshold (**a**) and preference (**b**) in men and women.

The distribution of salty taste threshold and preference in men and women are presented in Figure 1. We found that both salty taste threshold and preference were significantly different between men and women (*p* < 0.05).

The distributions of salty taste threshold and preference between men and women were significantly different as analyzed by the Cochran-Mantel-Haenszel test (*p* < 0.05).

The frequencies of the αENaC AA, AT and TT genotypes were 34.6%, 48.1% and 17.3% in men and 35.9%, 51.5% and 12.6% in women, respectively. The genotype distribution was not significantly different between men and women (*p* = 0.64). In both sexes, the major allele was the A allele, with a frequency of 0.59 in men and 0.62 in women (Table 2). No differences were found in either salty taste threshold or preference among the three genotype groups.

**Table 2:** Salty taste threshold and preference and zinc intake by αENaC A663T genotype in men and women ^a^.

αENaC A663T Genotypes	AA	AT	TT	*P* ^c^
***Men***				
*n* (%) ^b^	36 (34.6)	50 (48.1)	18 (17.3)	
Frequency of A allele	0.59			
Salty taste threshold (mM)	20.3 ± 12.0	18.6 ± 12.6	19.0 ± 11.0	0.58
Salty taste preference (mM)	81.9 ± 40.0	89.3 ± 40.0	85.0 ± 45.1	0.83
Zinc intake (mg/1000 kcal)				
Total zinc	4.3 ± 0.2	4.2 ± 0.1	4.0 ± 0.2	0.25
Available zinc	1.9 ± 0.1	1.8 ± 0.0	1.8 ± 0.1	0.32
***Women***				
*n* (%) ^b^	37 (35.9)	53 (51.5)	13 (12.6)	
Frequency of A allele	0.62			
Salty taste threshold (mM)	18.0 ± 11.5	14.7 ± 7.5	14.0 ± 6.1	0.28
Salty taste preference (mM)	71.7 ± 31.7	79.8 ± 28.3	67.6 ± 31.2	0.69
Zinc intake (mg/1000 kcal)				
Total zinc	4.5 ± 0.2	4.2 ± 0.1	4.7 ± 0.4	0.94
Available zinc	2.0 ± 0.1	1.9 ± 0.0	2.0 ± 0.2	0.93

^a^ Data are expressed as means ± SEM or *n* (%); ^b^ The αENaC genotypes distribution was not significantly different between men and women by χ^2^-test (*p* = 0.64); ^c^ Trends of change in variables of men or women across αENaC A663T genotypes were examined by GLM.

In Table 3, subjects were divided into three groups based on zinc intake, and the salty taste threshold and preference in each tertile was compared. In women, salty taste threshold was significantly lower (12.8 ± 1.0 mM) in the third tertile of total zinc intake than in the first tertile (17.1 ± 1.9 mM) (*p* = 0.04). Similarly, the salty taste threshold was significantly lower (12.2 ± 1.0 mM) in the third tertile of available zinc intake than in the first tertile (17.6 ± 1.9 mM) (*p* = 0.02). Correlation analyses showed that available zinc intake was negatively correlated with salty taste threshold in women (*r* = −0.21, *p* = 0.04) (data not shown). In men, salty taste thresholds were similar in all tertiles of available zinc intake.

We further examined the association between salty taste threshold and zinc intake by the αENaC A663T genotype (Table 4). In men, there was no detectable effect of zinc intake on salty taste threshold regardless of the αENaC A663T genotype. However, in women with the AA genotype, the higher intakes of total zinc or available zinc were related with lower salty taste threshold. In particular, a strong inverse association was found between available zinc intake and salty taste threshold (β = −0.833, *p* = 0.02) in women AA homozygotes.

**Table 3:** Salty taste threshold and preference by tertiles of zinc intake in men and women ^a^.

	Zinc Intake					*P* ^b^
	Tertile 1 (lowest)		Tertile 2		Tertile 3 (highest)	
***Total zinc***						
***Men***						
Threshold (mM)	20.8 ± 2.4		19.7 ± 1.8		17.2 ± 2.0	0.44
Preference (mM)	85.08 ± 7.9		93.7 ± 6.1		79.1 ± 6.5	0.36
***Women***						
Threshold (mM)	17.1 ± 1.9		17.6 ± 1.5		12.8 ± 1.0	0.04
Preference (mM)	77.2 ± 5.7		76.6 ± 4.6		72.5 ± 5.1	0.63
***Available zinc***						
***Men***						
Threshold (mM)	20.6 ± 2.2		18.7 ± 2.0		18.5 ± 1.9	0.73
Preference (mM)	82.1 ± 8.2		89.3 ± 6.4		86.6 ± 6.1	0.49
***Women***						
Threshold (mM)	17.6 ± 1.9		17.5 ± 1.5		12.2 ± 1.0	0.02
Preference (mM)	79.8 ± 5.4		72.6 ± 4.9		73.6 ± 5.2	0.60

^a^ Data are expressed as means ± SEM; ^b^ Trends of change in salty taste threshold and preference of men or women across tertiles of zinc intake were examined by GLM.

**Table 4:** Association between salty taste threshold and zinc intake according to αENaC A663T polymorphism in men and women ^a^.

Zinc Intake	αENaC A663T Genotype						
	AA				AT or TT		
	β	SE	*p*		β	SE	*p*
***Men***							
Total Zinc	0.040	0.50	0.94		−0.947	0.51	0.07
Available Zinc	0.038	0.40	0.93		−0.731	0.47	0.13
***Women***							
Total zinc	−0.765	0.41	0.07		−0.190	0.32	0.56
Available zinc	−0.833	0.35	0.02		−0.261	0.28	0.36

^a^ Multiple regression analysis models of predicting salty taste threshold with zinc intake by the αENaC A663T genotype.

**Figure 2:**
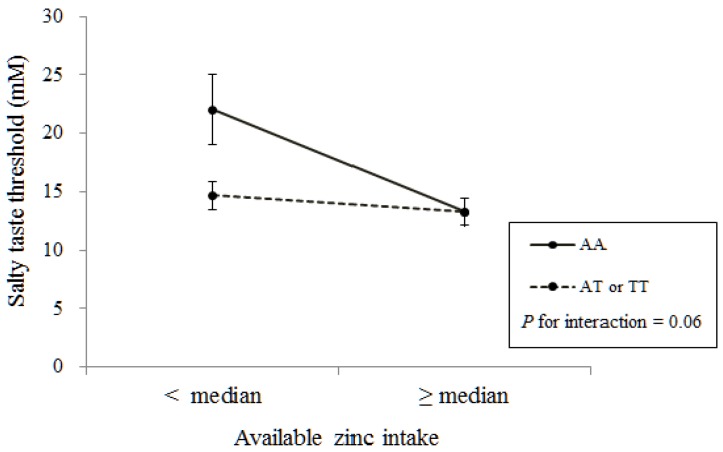
The joining effects of αENaC A663T genotypes and available zinc intake on salty taste threshold in women.

Examination regarding the joining effects of αENaC A663T genotypes and available zinc intake revealed that, for the AA genotype, salty taste threshold was significantly higher in women whose available zinc intake was below the median value than in women with higher zinc intake (Figure 2). In contrast, for the AT or TT genotype, the salty taste threshold was not significantly different between the two groups. There were marginally significant gene-nutrient interactions between αENaC A663T genotypes and available zinc intake in women (*p* = 0.06; Figure 2).

The interaction between available zinc intake and αENaC A663T genotypes were examined by GLM.

## 4. Discussion

In the present study, we examined the relationships among the αENaC A663T genetic polymorphism, zinc intake, and salty taste acuity and preference in young adults. We found that available zinc intake as well as total zinc intake was significantly associated with salty taste threshold in women. No relationship was found between αENaC A663T polymorphisms and either salty taste threshold or preference. However, the αENaC A663T polymorphisms modified the association between salty taste threshold and available zinc intake in women, suggesting that gene-nutrient interactions between the αENaC genotype and zinc intake play an important role in determining salty taste acuity.

Previous studies showed results consistent with our findings. In patients with taste impairment, serum zinc concentration was below normal value [21], and salivary zinc was lower in comparison to healthy subjects [36]. Also, Stewart-Knox *et al.* [27] found that subjects with higher erythrocyte zinc concentrations had a greater sensitivity for salt in a European elderly population. Further, a double-blind, randomized controlled intervention trial showed that the salty taste acuity of an elderly population aged 70–87 years was significantly increased by zinc supplementation at 30 mg zinc per day [20]. Similar to our study subjects’ age group, in young women aged 20–40 years, zinc intake was also positively associated with a taste acuity to salt [37]. These studies, however, did not examine the potential effects of genetic variations on the association between zinc status and salty taste perception.

The present study showed that three genotypes of the αENaC A663T polymorphism were found and the A allele was the major allele regardless of gender in Korean adults as observed in other ethnic groups. The mean frequency of the A allele in our study subjects was 0.60, and it is comparable to the frequency found in other Asian populations, including Japanese (0.58) [15]. A previous study in mice showed that amiloride-sensitive NaCl taste response was associated with genetic variation in the ENaC α-subunit [16]. However, in our study, no significant difference was observed in either salty taste threshold or salty taste preference based on the αENaC A663T genotype. Because ENaC consists of at least three subunits (α-, β- and γ-), genetic variations in the β- or γ- subunit may influence the salty taste perception [38]. Some genetic variants of the ENaC β-subunit, such as βENaC T594M and βENaC G442V, have been associated with functional changes in ENaC activity [39]. However, there βENaC variants appear to be very common in subjects of African origin but quite rare in white and Asian subjects [14,15]. Therefore, βENaC is unlikely to confer major genetic variability in salty taste perception, at least in Asian populations. Furthermore, some other taste receptors may be involved in salty taste perception. While ENaC is the only sodium-specific taste receptor identified to date, a nonselective cation taste receptor called TRPV1, a variant of the vanilloid receptor-1, has been proposed to play a role in salty taste perception because it responds to various cations including Na^+^, K^+^, NH^4+^ and Ca^2+^ [9]. Our study results warrant further genetic studies investigating both ENaC and TRPV1 genetic variations in a larger group of study subjects.

Most importantly, we found that available zinc intake was inversely associated with salty taste threshold only in women with the αENaC AA genotype (β = −0.833, *p* = 0.02). In contrast, no such association was found in women with the AT or TT genotype. The molecular mechanisms underlying this finding remain to be elucidated. Previous studies have reported that the αENaC A663T polymorphism is located in the distal COOH terminus of the α-subunit of ENaC and modifies its activity; compared to the A663 form, the T663 form exhibited increased surface expression, which was associated with significantly higher currents [40,41,42]. These results suggest that the αENaC AA homozygotes possess lower ENaC activity than αENaC T663 allele carriers do. Therefore, it is likely that the optimal zinc intake to ensure salty taste acuity is more crucial in AA homozygotes, which is well corroborated by our study findings.

Unlike women, no association was found between zinc intake and salty taste threshold in men. In our study, the salty taste threshold was significantly lower in women than in men, which is consistent with previous studies showing that women are more sensitive to salty and other basic (sweet, sour and bitter) tastes than men [37,43,44]. Anatomical data have shown that the density of fungiform papillae and taste bud cells is higher in women than in men, and supertasters tend to have more of these taste receptor cells [45]. Because zinc is required for the activity of gustin, which in turn is necessary for the development of taste receptor cells [18], it is possible that women are more susceptible to the changes in zinc status and associated taste function. Further studies are needed to clarify the mechanism(s) of the sex differences in terms of the role of zinc on salty taste acuity.

In the present study, we examined the association of salty taste perception with zinc intake. Alternatively, serum zinc is also commonly used to estimate zinc status. But, serum zinc has limitations as it is under homeostatic control and is relatively stably maintained until severe dietary zinc restriction occurs (<2–3 mg/day). In addition, factors such as stress, infection, and inflammation are known to affect serum zinc [46]. As a result, the association between serum zinc and zinc intake has been weak in observational studies on free-living subjects [47,48]. Our data in the current study also showed that serum zinc levels were not significantly correlated with either total or available zinc intake (*p* > 0.05) (data not shown). Since a major factor associated with the development of zinc deficiency in population is inadequate intakes of zinc [48], we used total and available zinc intakes as indicators of zinc status in the current study.

No association between zinc status and salty taste preference was found in this study. It has been suggested that taste preference can be affected by various factors, including socioeconomic variables as well as behavioral and physiologic factors [49]. Therefore, further comprehensive study of the relationships between salty taste preference and zinc status, including other associated factors, is needed.

## 5. Conclusions

In summary, we found a gender-specific association between salty taste acuity and available zinc intake in young adults. Moreover, we demonstrated that the association between salty taste acuity and available zinc intake is more evident in women with αENaC AA genotypes. These results provide novel evidence for the gene-nutrient interactions affecting the salty taste perception. From a public health perspective, our findings implicate that the information on the αENaC A663T gene polymorphisms may improve predictions of a potential risk group for excessive sodium intake and help to identify individuals who may benefit the most from adequate zinc intake. Further studies are needed to strengthen these results in a larger population as well as in other ethnic groups, and to establish dietary guidelines to reduce salt intake, depending on the αENaC genotypes and the gender of the target population.
